# The impact of biologic therapies on extra-intestinal manifestations in inflammatory bowel disease: A multicenter study

**DOI:** 10.3389/fmed.2022.933357

**Published:** 2022-08-08

**Authors:** Francesca Ferretti, Maria Camilla Monico, Rosanna Cannatelli, Stefania Carmagnola, Marco Vincenzo Lenti, Antonio Di Sabatino, Francesco Conforti, Luca Pastorelli, Flavio Caprioli, Cristina Bezzio, Simone Saibeni, Stefano Mazza, Maurizio Vecchi, Giovanni Maconi, Sandro Ardizzone

**Affiliations:** ^1^Gastroenterology Unit, ASST Fatebenefratelli-Sacco, Department of Biomedical and Clinical Sciences, University of Milan, Milan, Italy; ^2^Department of Internal Medicine, Fondazione IRCCS Policlinico San Matteo, Clinica Medica, Università degli Studi di Pavia, Pavia, Italy; ^3^Gastroenterology and Endoscopy Unit, Fondazione IRCCS Ca’ Granda, Ospedale Maggiore Policlinico di Milano, Milan, Italy; ^4^Department of Pathophysiology and Transplantation, University of Milan, Milan, Italy; ^5^Gastroenterology Unit, IRCCS Policlinico San Donato, San Donato Milanese, Italy; ^6^Department of Health Sciences, University of Milan, Milan, Italy; ^7^Gastroenterology Unit, ASST Rhodense, Rho Hospital, Rho, Italy; ^8^Gastroenterology and Digestive Endoscopy Unit, ASST di Cremona, Cremona, Italy

**Keywords:** inflammatory bowel disease, extra-intestinal manifestations (EIMs), biologic therapy, Vedolizumab, TNF inhibitors, Ustekinumab

## Abstract

**Introduction:**

Patients with inflammatory bowel disease (IBD) have a high risk of developing extra-intestinal manifestations (EIMs). We aimed to assess the cumulative incidence and clinical course of EIMs in patients treated with Vedolizumab (VDZ) and non-gut selective biologic drugs.

**Materials and methods:**

In this multicenter observational study, we enrolled 1,182 patients with IBD under biologic treatment in tertiary care centers, collecting the rate of new-onset EIMs and the clinical course of new and pre-existing EIMs since the introduction of the ongoing biologic drug (259 VDZ vs. 923 non-gut selective agents, median time 3 vs. 4 years).

**Results:**

Among 1,182 patients with IBD (median age of 46 years; 55% men) on biologics, the overall cumulative incidence of new onset EIMs was 4.1% (49/1,182), in particular 6.6% (17/259) on VDZ vs. 3.5% (32/923) on non-gut selective biologics (*p* = 0.02). Among 224 patients reporting new or pre-existing EIMs, those on VDZ showed a higher rate of clinical worsening compared with non-gut selective therapies (15.5 vs. 7.3%, *p* = 0.08). However, both showed a similar rate of modification of the therapeutic regimen. Female gender [hazard ratio (*HR*) 2.18], a longer course of ongoing biologic therapy (*HR* 1.18), ulcerative colitis (UC) (*HR* 1.83), and VDZ therapy (*HR* 1.85) were significant risk factors for developing new EIMs.

**Discussion:**

Our study suggests that the type of biologic treatment might affect the risk of developing EIMs, with a slightly higher risk in patients on gut-selective therapies. However, a similar clinical course is observed in the two groups.

## Introduction

Both ulcerative colitis (UC) and Crohn’s disease (CD) have a high risk of developing extra-intestinal manifestations (EIMs) since almost 40% of patients will develop an EIM during the course of gastrointestinal disease (1–3). EIMs may affect different organs of the musculoskeletal, skin, ocular, and hepatobiliary system, accounting for a relevant clinical problem, (4) that may occur both during clinical activity and remission phases of an intestinal disease (4, 5).

Since the high prevalence of EIMs in inflammatory bowel diseases (IBD) and their negative influence on patients’ quality of life and the healthcare system, assessment of EIMs should be undertaken on a regular basis during the follow-up of these patients to ensure adequate treatment.

The main goals of IBD medical treatment are the induction and maintenance of clinical and endoscopic remission to treat symptoms, guarantee an improved quality of life, and prevent complications leading to hospital admission and surgery. Currently, medications used to treat IBD include various agents which are tailored based on treatment indication, disease extent, and severity. Since the introduction of the first biologic agents, the therapeutic scenario has deeply evolved. Biologics were initially restricted to multi-failure patients who had already experienced mesalamine, corticosteroids, or immunosuppressants, such as azathioprine, in a so-called “step-up” therapy. Actually, an early approach with biologic agents with a “top-down” therapeutic strategy has shown substantial benefit in the management of selected patients with moderate-to-severe IBD, both in terms of clinical and endoscopic outcomes (6).

Biologic drugs currently available are monoclonal antibodies that target different inflammatory pathways, such as antibodies against tumor necrosis factor-alpha (anti-TNF-α), interleukin 12/23, anti-integrins, and small molecules Janus kinase (JAK) inhibitors, such as tofacitinib. Choosing which biologic is the most appropriate for each individual patient may sometimes be challenging, especially in presence of concomitant EIMs.

Vedolizumab (VDZ), a monoclonal antibody against α_4_β_7_ integrin, acts by preventing leucocyte migration and homing toward the gut mucosa and has proven its efficacy as induction and maintenance therapy in both UC and CD (7, 8). The specific gut-selective effect of VDZ makes its safety profile extremely favorable (9). Conversely, its action may not influence the course of EIMs (8, 10, 11). Indeed, data on the efficacy of VDZ on EIMs are scarce and often discordant (12). A recent study by Dubinsky et al. suggests that treatment with VDZ may actually increase the likelihood of developing *de novo* EIMs (13).

Therefore, we aimed to analyze the incidence of new onset EIMs and the clinical course of new and pre-existing EIMs comparing patients treated with VDZ with those on non-gut selective biologic drugs in a large cohort of patients with IBD under biologic therapy.

## Materials and methods

We retrospectively collected data about all the adult patients with IBD on biologic therapy in their regular clinical follow-up at 6 tertiary referral centers in Lombardy.

Eligible patients were adults (>18 years old) with a confirmed diagnosis of IBD (CD, UC, and undetermined IBD) under treatment with any of the currently available biologic therapies for at least 2 months. All patients had a periodic and updated follow-up visit in the previous months.

Demographical and clinical data (age, gender, IBD type, ongoing and previous biologic therapies, smoking status, and the presence of EIMs before the start of the ongoing treatment) were retrieved from medical records.

According to the European Crohn’s and Colitis Organization (ECCO) guidelines, (14) the main EIMs included were: rheumatologic (peripheral and axial arthropathies), mucocutaneous (stomatitis, pyoderma gangrenosum, erythema nodosum, and psoriasis), ophthalmologic (episcleritis and uveitis), hepatobiliary [primary sclerosing cholangitis (PSC)], and others (such as pancreatitis and central nervous system manifestations).

The diagnosis of EIMs was confirmed by other specialists’ medical reports (rheumatologists, ophthalmologists, dermatologists, and hepatologists) and/or objective data from imaging, histology, and laboratory tests.

Data about the onset and the clinical course of EIMs were retrospectively collected and retrieved from medical records.

We included the “new onset” EIMs (intended as any EIMs occurred after the introduction of the ongoing biologic therapy) and “pre-existing” EIMs (intended as any EIMs already mentioned before the introduction of the ongoing biologic therapy).

The course was defined as improvement or worsening of EIM-related symptoms during the follow-up. In the case of clinical worsening, we assessed the need to modify the therapeutic regimen by introducing an adjunctive therapy (corticosteroids/anti-inflammatory drugs/immunomodulators) or by switching/optimizing the ongoing biologic treatment.

The primary endpoints of this study were to assess the cumulative incidence of new onset EIMs in two cohorts of patients with IBD on biologic treatment (gut selective vs. non-gut selective) in clinical follow-up since the start of each treatment and to identify any potential risk factor for developing new EIMs.

The secondary endpoint of this study was to assess the clinical course of new onset and pre-existing EIMs in these two cohorts of patients. In particular, we aimed to analyze whether VDZ was associated with a higher incidence of *de novo* EIMs or with the clinical worsening of pre-existing EIMs, needing adjunctive and/or switching therapy.

Data were inserted into a database accessible to all participating centers. This study was an observational, retrospective study, using de-identified data from medical records and the research was conducted according to the principles of the Declaration of Helsinki; therefore, it was exempted from the Institutional Review Board approval. The data underlying this article will be shared upon reasonable request to the corresponding author.

### Statistical analysis

Demographic and clinical data were expressed as numbers or percentages for discrete variables and as mean and standard deviation (SD) or median and interquartile range (IQR) for continuous variables, according to their distribution.

The prevalence of EIMs was defined as the “number of persons with EIMs/overall population” at the time of the data collection.

The cumulative incidence of EIMs was defined as the “number of new onset cases/overall population” since the introduction of the ongoing biologic drug.

The gut-selective (VDZ) and non-gut selective groups were compared with the chi-square or Fisher’s exact test for categorical variables and the Mann–Whitney analysis for continuous variables. A *p*-value of < 0.05 value was considered statistically significant.

When variables were not available for some patients, these were excluded for percentage calculation. Univariate and multivariate analyses with logistic regression were performed, and hazard ratio (*HR*) was calculated.

Statistical analyses were done using IBM SPSS Statistics (release 23; IBM corporation, United States).

## Results

We retrospectively collected data on 1,182 patients with IBD (797 CD and 385 UC) in clinical follow-up on treatment with biologics. Demographic and clinical data are shown in Table 1.

**TABLE 1:** Demographic and clinical parameters of included patients.

	Overall	Gut-selective therapies^1^	Non-gut selective therapies^2^	*P*
*N*	1182	259	923	–
Age, mean ± SD	46 ± 15	52 ± 17	45 ± 14	<0.01
Male pts, *n* (%)	653 (55)	141 (54)	512 (56)	0.77
IBD, *n* (%)				
CD UC	797 (67) 385 (33)	105 (41) 154 (59)	692 (75) 231 (25)	<0.01
Smokers, *n* (%)*	305 (26)	56 (25)	249 (32)	0.21
Disease duration, years (mean ± SD)	14 ± 9	14 ± 9	14 ± 9	0.98
Ongoing biologic treatment duration, years (mean ± SD)	4 ± 2	3 ± 1	4 ± 3	<0.01
Previous biologic therapy, n (%)	535 (45)	162 (63)	305 (33)	<0.01

*Data not available are excluded from the calculation.

^1^ Vedolizumab,

^2^ Infliximab, Adalimumab, Golimumab, Ustekinumab.

**FIGURE 1:**
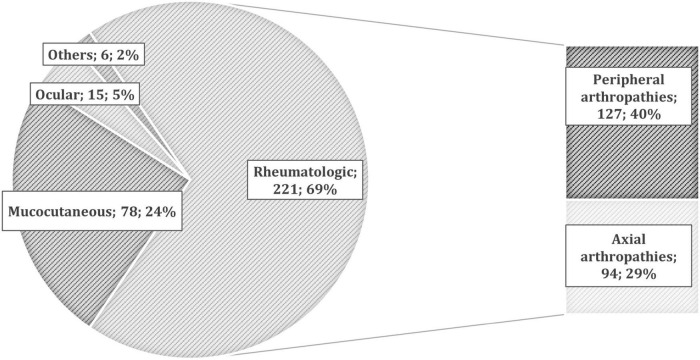
The prevalence of different extra-intestinal manifestations (EIMs) in patients with inflammatory bowel disease (IBD). According to the European Crohn’s and Colitis Organization (ECCO) definitions (11), we collected 221 rheumatologic EIMs (127 peripheral and 94 axial arthropathies), 78 mucocutaneous (10 pyoderma gangrenosum, 36 erythema nodosum, 21 psoriasis, and 11 others), 15 ocular (6 episcleritis and 9 uveitis), and 6 others [such as primary sclerosing cholangitis (PSC), pancreatitis, and central nervous system manifestations].

The overall prevalence of patients with at least one EIM in our IBD cohort was 19.8% (234/1,182) (Figure 1), including both the pre-existing EIMs and the ones which developed after starting the last biologic treatment. They were 44% men, 65% CD, and 35% UC, with a mean age of 51 ± 14 years. Of them, about one-third (79/234) reported multiple concomitant EIMs.

Patients were under treatment with different biologic agents: 307 on intravenous anti-TNF (Infliximab), 505 on subcutaneous anti-TNF (Adalimumab, Golimumab), 111 on Ustekinumab, and 259 on Vedolizumab. The median duration of the ongoing therapy was 4 years (with a range of 1–16 years). In this follow-up period, the incidence of new onset EIMs was 4.1% (49/1,182): 33 rheumatic (5 axial, 26 peripheral, and 2 both axial and peripheral arthropathies), 14 cutaneous (10 cases of psoriasis, 2 cases of aphthous stomatitis, one case of pyoderma gangrenosum, and one case of suppurative hidradenitis), one case of idiopathic pancreatitis, and one case of autoimmune hemolytic anemia.

The overall incidence of new EIMs in patients on treatment with VDZ was statistically higher compared with patients under non-gut selective therapies (6.6 vs. 3.5%, 17/259 vs. 32/923, *p* = 0.02). Interestingly, this difference mainly depends on the highest incidence of rheumatic diseases among patients in the gut selective group (4.6 vs. 2.4%, 12/259 vs. 22/923, *p* = 0.05), while the incidence of cutaneous diseases was comparable in the two cohorts (1.9 vs. 1.1%, 5/259 vs. 10/923, *p* = 0.4).

According to the univariate analysis, older and female patients, suffering from UC, with a longer course of biologic therapy, and under treatment with gut-selective agents showed a higher risk of developing a new EIM (Table 2). In the multivariate analysis, only the female gender and the duration of the ongoing biologic treatment maintained statistical significance (Table 2).

**TABLE 2:** The univariate and multivariate analysis of clinical variables predicting the new onset of extra-intestinal manifestations (EIMs) in our cohort of patients with inflammatory bowel disease (IBD).

			Univariate analysis			Multivariate analysis		
	Cases of new EIMs	Controls	HR	95% CI	P	HR	95% CI	P
N	49	1133	–	–	–			
Age, mean ± SD	51 ± 14	46 ± 15	1.02	1.00–1.04	**0.02**	1.02	1.00–1.04	0.10
Female pts, *n* (%)	31 (63)	498 (44)	2.20	1.21–3.97	**0.009**	2.18	1.19–3.98	**0.01**
IBD type, *n* (%) UC	23 (47)	362 (32)	1.89	1.06–3.35	**0.03**	1.83	0.98–3.41	0.06
Smokers, *n* (%)*	15 (34)	290 (30)	1.2	0.64–2.29	0.60			
Disease duration, median (IQR)	15 ± 10	14 ± 9	1.01	0.98–1.05	0.58			
Type of biologic, *n* (%) VDZ	17 (35)	242 (21)	1.96	1.07–3.58	**0.03**	1.85	0.91–3.72	0.08
Duration of ongoing biologic, median (IQR)	5 ± 3	4 ± 2	1.15	1.04–1.27	**0.005**	1.18	1.07–1.31	**0.002**
Previous EIM, *n* (%)	9 (18)	185 (16)	1.15	0.55–2.41	0.71			
Previous biologic treatment, *n* (%)	19 (39)	449 (43)	0.95	0.53–1.71	0.87			

*Data not available are excluded from the calculation. Bolded values represent statistically significant <0.05 values.

In the whole cohort of patients, 194 patients reported at least one EIM even before the start of the ongoing treatment. Indeed, about one-third of patients (66/194, 34%) were already on therapy with an ongoing adjunctive treatment, including 35 patients on steroids (topical or systemic), 11 on methotrexate (MTX), 9 on salazopyrin (SASP), 9 on analgesics, and 2 on other therapies (hydroxychloroquine and ciclosporin). Independent of biologic agents, of 20 patients with pre-existing EIMs already on disease-modifying antirheumatic drugs (DMARDs), one patient (5%) developed an EIM flare, compared with 6.9% (12/174) of patients who were not taking DMARDs (*p* = 0.7). Globally, we retrieved the clinical course of the 224 patients reporting a new or pre-existing EIM since the introduction of the ongoing biologic therapy. Of them, a clinical improvement was observed in about 90% of patients (204/224, partial in 52, and complete in 152), while a worsening of disease was reported in 20 patients.

Patients on VDZ showed a higher rate of clinical worsening since the introduction of the biologic agent compared with non-gut selective therapies, even if the results did not reach statistical significance (15.5 vs. 7.3%, 7/45 vs. 13/179, *p* = 0.08). This trend of worsening was observed mainly in the case of rheumatic EIMs (18.5 vs. 6.9%, 5/27 vs. 9/131, *p* = 0.05), while this association was not observed in cutaneous EIMs (7.7 vs. 6.2%, 1/13 vs. 3/48, *p* = 0.8). Thus, we analyzed the need for a modification of the ongoing therapeutic regimen to control the clinical worsening: over 80% of patients did not undergo any modification in both groups (87% of patients on VDZ vs. 81% on other treatments, 39/45 vs. 145/179, *p* = 0.1). Moreover, the rate of DMARDs addition or change/optimization of biologic therapy was comparable (4/45 vs. 25/179, *p* = 0.5, and 1/45 vs. 14/179, *p* = 0.2).

## Discussion

In the current scenario of IBD treatments, multiple biologic options are available with high and comparable levels of efficacy on IBD activity. Hence, choosing which biologic is the most appropriate for a specific patient can sometimes be a difficult task, especially in the presence of EIMs. In this regard, the available evidence on the efficacy of gut-selective therapies, such as VDZ on EIMs, is often conflicting and results only from case series, prospective or retrospective cohort studies, or the *post-hoc* analysis of randomized controlled trials (RCT) (13, 15, 16). To date, no comparative head-to-head RCTs between VDZ and non-gut selective biologics are available to define their efficacy in EIMs.

In a recent retrospective cohort study, Dubinsky et al. analyzed large databases of insurance claims and identified a 28% higher incidence of EIMs in patients with CD on VDZ compared with patients on anti-TNF agents. On the contrary, this effect was not statistically significant in patients with UC even if a higher incidence of aphthous stomatitis, pyoderma gangrenosum, and PSC was described. However, this study was limited by the use of ICD-9 and ICD-10 diagnosis codes and de-identified insurance claims data (13).

In our real-life multicenter study, based on a large cohort of patients with IBD on clinical follow-up in tertiary referral centers, we found a statistically higher incidence of new onset EIMs among patients on VDZ compared with patients on non-gut selective therapies, despite a shorter observation time. Including all available clinical variables, the univariate analysis showed a positive correlation between the risk of developing new EIMs and female sex, older age, IBD type, longer duration of current biologic treatment, and VDZ therapy. In multivariate analysis, female sex proved to be the strongest predictive factor for the onset of new EIMs (*HR* 2.18). Moreover, a slightly higher risk of developing new EIMs in patients with a longer course of the ongoing biologic therapy (*HR* 1.18), UC (*HR* 1.83), and VDZ therapy (*HR* 1.85) was observed. Instead, no correlation was observed between the risk of developing new EIMs and long duration of disease and smoking status. Moreover, neither a concomitant therapy with steroids and/or immunosuppressants nor a previous biologic treatment influences the risk of developing EIMs.

Musculoskeletal and cutaneous diseases are the most frequently observed EIMs. Regarding these types of EIMs, controversial data are available in the literature. A systematic review by Chateau et al. recently demonstrated that treatment with VDZ may have no effect on preexisting arthralgia and arthritis but it may play a role in reducing the incidence of new rheumatic manifestations compared with placebo (15). In our study, patients treated with VDZ showed a higher rate of worsening of pre-existing rheumatic EIMs, even though the trend did not reach statistical significance. Moreover, the need for adjunctive therapy (as DMARDs) or withdrawal/change of biologic agents was similar between the two treatment groups. Indeed, in most cases, the clinical worsening did not require a major modification of the maintenance therapy. Patients’ symptoms were managed with on-demand analgesic or anti-inflammatory drugs in case of rheumatic manifestations or topical agents in the case of cutaneous manifestations.

These results are in line with Ramos et al., who reported that almost one-third of 201 patients under VDZ had a worsening of preexisting EIMs, and peripheral arthritis was the most affected (17). In addition, in the multicenter cohort study by the GETAID OBSERV-IBD, about 14% of patients developed arthralgia (16). On the contrary, in a *post-hoc* analysis of the GEMINI Trials, long-term treatment with VDZ was found to be associated with a reduced incidence of worsening/new arthralgia and arthritis (18). This effect could be explained, especially in the EIM linked to the activity of the disease, by the intestinal remission of the disease induced by VDZ.

Cutaneous manifestations seem to be less affected by the introduction of VDZ: in particular, in the OBSERV-IBD study, up to 75% of cutaneous EIMs were in remission after 54 weeks of VDZ (13). In addition, Ramos et al. reported stability of disease in 77% of cutaneous EIMs despite the introduction of VDZ as biologic therapy (17). Similarly, according to our study, the effect of VDZ on the incidence of new onset EIMs was observed only in rheumatic manifestations, since the incidence of cutaneous manifestations was not statistically different between the two treatment groups.

Furthermore, in the case of previous biologic treatment, the cumulative effect of multiple therapeutic lines on pre-existing EIMs was difficult to retrieve and analyze. However, in multivariate analysis, previous biologic treatment did not demonstrate an impact on the risk of developing EIMs.

To our knowledge, this is the first multicenter study that evaluated the cumulative incidence of new onset EIMs after VDZ initiation in a very large cohort of patients with IBD, all in clinical follow-up at tertiary referral IBD units. Moreover, we evaluated the clinical course of EIM in this very large cohort of patients with IBD under biological therapy.

Our study was limited by the retrospective design, making it difficult to collect data regarding the activity of intestinal inflammation at the time of EIM occurrence. Nonetheless, when analyzing the incidence of new rheumatic EIMs by the proportion of axial vs. peripheral arthropathy, the latter typically following intestinal disease activity, no difference was found between the two treatment groups. To clarify this point, future prospective and targeted studies should be performed.

Finally, the cohort included patients with IBD with different follow-ups. As expected, the time of exposure to anti-TNF was superior to VDZ as it is available for a longer time. However, it is noteworthy that, even after a shorter time of exposure, higher rates of EIM onset were observed in the gut-selective cohort compared with the non-gut-selective cohort.

In conclusion, our study suggests that the type of biologic treatment may have an impact on the risk of developing *de novo* EIMs, especially rheumatologic manifestations. Thus, in patients presenting concomitant risk factors for EIMs, if possible, therapeutic strategies other than VDZ should be taken into consideration as the first-line approach. Otherwise, in the case of VDZ treatment, it is advisable to closely monitor for the occurrence of rheumatic symptoms, which may prompt further workup and/or adjunctive therapies. Of course, the design of specific RCTs and prospective studies is advisable for offering more robust evidence in the future.

## Data availability statement

The raw data supporting the conclusions of this article will be made available by the authors, without undue reservation.

## Ethics statement

Ethical review and approval was not required for the study on human participants in accordance with the local legislation and institutional requirements. Written informed consent for participation was not required for this study in accordance with the national legislation and the institutional requirements.

## Author contributions

SA, FF, MM, and GM: planning the study, drafting the article, data collection, and interpretation. AD, CB, FCa, FCo, LP, ML, MV, RC, SS, SC, and SM: data collections and critical revision of the manuscript for important intellectual content. All authors approved the final version of the manuscript including the authorship list.
